# Head transcriptome profiling of glossiphoniid leech (*Helobdella austinensis*) reveals clues about proboscis development

**DOI:** 10.1098/rsob.210298

**Published:** 2022-03-02

**Authors:** Hee-Jin Kwak, Sung-Gwon Lee, Soon Cheol Park, Jung-Hyeuk Kim, David A. Weisblat, Chungoo Park, Sung-Jin Cho

**Affiliations:** ^1^ Department of Biological Sciences and Biotechnology, College of Natural Sciences, Chungbuk National University, Cheongju, Chungbuk 28644, Republic of Korea; ^2^ Department of Ecology, Evolution and Behavior, Alexander Silberman Institute of Life Sciences, Faculty of Science, Hebrew University of Jerusalem, Jerusalem 9190401, Israel; ^3^ School of Biological Sciences and Technology, Chonnam National University, Gwangju 61186, Republic of Korea; ^4^ Department of Life Science, Chung-Ang University, Seoul 06974, Republic of Korea; ^5^ Wildlife Disease Response Team, National Institute of Wildlife Disease Control and Prevention, Incheon 22689, Republic of Korea; ^6^ Department of Molecular and Cell Biology, University of California, 385 Weill Hall, Berkeley, CA 94720-3200, USA

**Keywords:** leech, proboscis, *de novo* transcriptome assembly, differentially expressed genes

## Abstract

Cephalization refers to the evolutionary trend towards the concentration of neural tissues, sensory organs, mouth and associated structures at the front end of bilaterian animals. Comprehensive studies on gene expression related to the anterior formation in invertebrate models are currently lacking. In this study, we performed *de novo* transcriptional profiling on a proboscis-bearing leech (*Helobdella austinensis*) to identify differentially expressed genes (DEGs) in the anterior versus other parts of the body, in particular to find clues as to the development of the proboscis. Between the head and the body, 132 head-specific DEGs were identified, of which we chose 11 to investigate their developmental function during embryogenesis. Analysis of the spatial expression of these genes using *in situ* hybridization showed that they were characteristically expressed in the anterior region of the developing embryo, including the proboscis. Our results provide information on the genes related to head formation and insights into the function of proboscis-related genes during organogenesis with the potential roles of genes not yet characterized.

## Graphical abstract

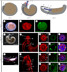

## 1. Introduction

In the development of bilaterian animals, a broadly conserved genetic toolkit underlies the specification of positional identity along the body axis, usually including the establishment of a centralized nerve system, concentration of sensory organs and development of specialized mouth parts at the most anterior region [1–3]. The mouth parts of animals have undergone tremendous specialization and diversification as they have evolved to capture and ingest food according to their diverse life styles and ecosystems [4–7], in large part by modifying the regulation and deployment of the broadly conserved toolkit. Invertebrates especially show a wide variety of mouthpart structures such as tooth, mandible/maxilla and proboscis, depending on diverse food sources, at the end of the head part [7–12]. Among various mouthpart structures in invertebrates, the organ called the proboscis has been known as a specialized apparatus for piercing and holding the host's gut wall [13], capturing prey through injecting toxin [14,15] and fluid sucking [16,17], and the proboscis as a feeding apparatus has a tube for sucking up food such as nectar, body fluid and cellular constituents [18,19]. Fluid sucking of insects is generated in the canalized proboscis by suction pressure produced from the proboscis–cranial sucking pump complex with capillary activity which directs the flow of fluid into the oesophagus [19–22]. On the other hand, the feeding tube of the leech, another representative proboscis-bearing animal, consists only of a muscle complex. The force generated by this complex induces peristalsis for moving food in an aboral direction [11,16].

Cell lineage experiments performed with a well-studied leech model, *Helobdella* (family Glossiphoniidae), have provided information on the development of the leech proboscis [23,24]. The formation of germ layer-specific tissues in the proboscis is achieved through orchestrated proliferation and interdigitation of clones arising from cells in the early lineage of the mesodermal proteoblast DM' (‘4d cell' in spiralian nomenclature), which mainly contribute to the formation of non-segmental mesoderm that can develop into the digestive tract during organogenesis [23]. At stage 10 (220 h to 245 h after zygotic development), the proboscis develops in an everted. During stage 11, the proboscis gradually inverts to its resting adult position within the foregut region, by which time it consists of a complex array of longitudinal, radial and circular muscles [23,25]. These radical and dynamic developmental changes raise the question of whether essential factors are actively expressed at stage 10 for the formation of the head or specific structures such as the proboscis.

Over the past few years, many studies have been performed to understand the develpomental involvement of various transcription factors [26–28] and signalling molecules [29–31], including physiological substances in the anterior region of the leech [32]. Previous studies have suggested that anterior development involves a combination of numerous signalling pathways and transcription factors and that the anterior head is a vast hub of gene expression. However, large-scale studies on expressed genes have not been reported. Towards this end, we used stage 10 leech embryos and conducted RNA sequencing (RNA-seq), a successful method for understanding differential gene expression in specific tissues or under different conditions in a wide variety of animals and plants [33–35]. In this study, we provide detailed information on the stage 10 leech embryo transcriptome and present proboscis-specific genes identified via differentially expressed gene (DEG) analysis between the head and body. Our results will be used as comparative data for the evolutionary relevance of the proboscis and also fill the gap in transcriptional information about the missing stage during leech embryo development.

## 2. Results and discussion

### 2.1. Internal structure development of the proboscis during organogenesis

**Figure 1.**
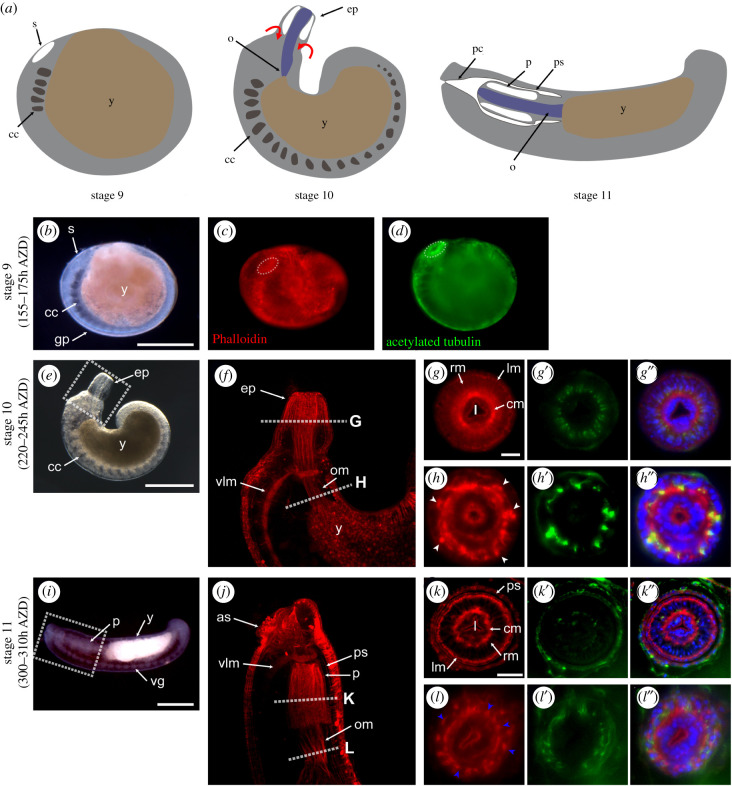
Proboscis formation and internal structure differentiation. The formation and internal structure differentiation of the leech proboscis. All embryos are oriented anterior to the left and dorsal to the top of the panel. White dotted rectangles indicate the magnified region in (*e*) and (*i*). White dotted lines indicate the sectional regions. (*a*) Schematic of organogenesis stages. Between stages 10 and 11, the proboscis invaginates (red arrows) into the mouth pore and locates in the foregut region. (*b*–*d*) At stage 9, the oral precursor stomodeum (white dotted circles) develops in the anterior region. (*e*,*f*) Lateral view of the stage 11 embryo showing the everted proboscis. (*g*–*h*) Sectioned views showing the primordium of innervated muscle fibres (white arrowheads). (*i*–*j*) Lateral view of the stage 11 embryo showing ganglia and the invaginated proboscis located in the foregut region. (*k*–*l*) Sectioned views showing differentiated muscles and nerve fibres (blue arrowheads). Scale bar, 200 µm in panels *b*, *e*, *i*; 20 µm in panels *g* and *k*. as, anterior sucker; AZD, after zygotic development; cc, coelomic cavity; cm, circular muscle; ep, everted proboscis; gp, germinal plate; l, lumen; lm, longitudinal muscle; o, oesophagus; om, oesophageal muscle; p, proboscis; pc, proboscis chamber; ps, proboscis sheath; rm, radial muscle; s, stomodeum; vg, ventral ganglion; vlm, ventral longitudinal muscle; y, yolk.

The harmonious proliferation and differentiation of the progeny of lineage-specific blast cells and micromeres give rise to the development of epidermis, ganglia and musculature with the formation of primary mouthparts that can develop into a future digestive tract [23–25,36]. After epiboly, the anterior germinal plate develops into the precursor of the mouthpart (stomodeum), surrounded by primary nerves and muscle fibres (figure 1*a–d*) [23]. At stage 10, the proboscis gradually protrudes, covered with epithelial tissues which develop into the proboscis sheath. This process involves the active development of a complex of innervated muscle layers in the proboscis structure and oesophagus (figure 1*a,e*–*h*″). By stage 11, the proboscis invaginates into the mouth pore located in the foregut region with placement of a presumptive circumferential muscle layer and spanning of the radial muscle along with development of longitudinal and oesophageal muscle (figure 1*a*,*i*–*l*′). The timing of the morphological differentiation of the proboscis thus suggests that stage 10 might be a crucial step in the development of the internal structure and that proboscis-related genes might be highly expressed during this dynamic event.

### 2.2. *De novo* transcriptome assembly of a stage 10 leech embryo

**Figure 2.**
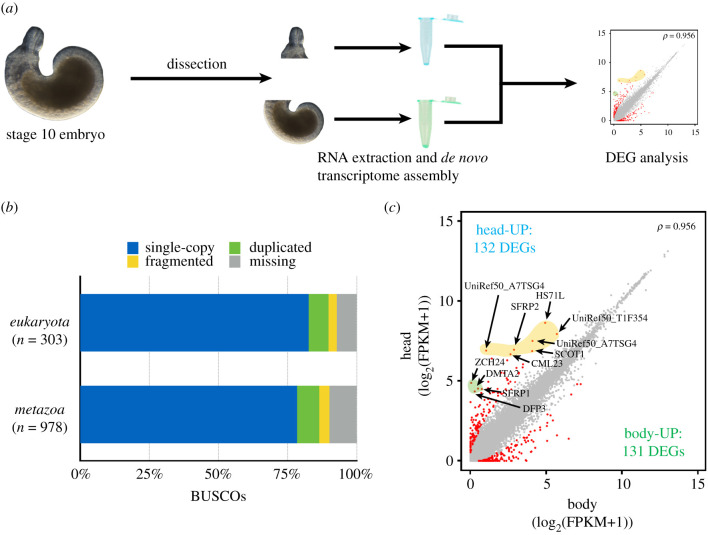
Schematic of the analysis and DEGs between the proboscis and body. (*a*) At stage 10, the head and body of embryos were cut into sections. Each section was used to obtain raw reads after RNA extraction followed by DEG analysis (for detailed flow information, see electronic supplementary material, figure S1). (*b*) Results of BUSCO analysis. (*c*) Scatterplot showing global gene expression profiles (log2-transformed FPKM+1) between the proboscis and body. Red, DEGs; yellow, highly expressed genes; green, specifically expressed genes. *ρ* is Spearman's correlation coefficient. FPKM**,** fragments per kilobase of transcript per million mapped reads.

We used RNA-seq to find specific genes in the proboscis compared with the body region at stage 10 (figure 2*a* and electronic supplementary material, figure S1). More than 85 million raw reads of the body and 79 million raw reads of the proboscis were obtained, yielding 84 million and 78 million filtered reads, respectively, with high-quality values (filtered-out ratio greater than 98%; *Q*_20_ > 98%; for details, see electronic supplementary material, table S1). We combined these reads and subjected them to *de novo* transcriptome assembly using Trinity v. 2.1.1. The transcriptome assembly yielded 127 484 transcripts with an *N*_50_ length of 1002 bp and an average length of 655.1 bp (for details, see electronic supplementary material, table S1). Subsequently, we annotated the assembled transcripts using BLASTP (e-value ≤1 × 10^−10^) based on Uniprot/SwissProt and UniRef50 databases to obtain complete sequence coverage. We obtained 13 735 annotated transcripts, including 8003 genes annotated based on the Uniprot/SwissProt database and 5732 genes annotated based on the UniRef50 database (electronic supplementary material, tables S1 and S2). To examine the completeness of this *Helobdella austinensis* embryo transcriptome, we assessed annotated protein-coding sequences. A total of 82.5% (250/303 genes) and 78.4% (767/978 genes) of the eukaryote and metazoan single-copy orthologues were identified, respectively (figure 2*b*).

### 2.3. Identification of differentially expressed genes and gene ontology analysis

Of the 13 735 annotated transcripts, 263 were DEGs in the head and the body (false discovery rate [FDR] < 0.05) (figure 2*c*). Of these 263 DEGs, 132 and 131 were specifically upregulated in the head and the body, respectively (electronic supplementary material, tables S1 and S3). Characteristically, more than half of these DEGs were uncharacterized genes covered by Uniprot reference clusters [37] (70 of 132), suggesting that anterior development involved a larger proportion of putatively leech-specific genes during anterior formation.

**Figure 3.**
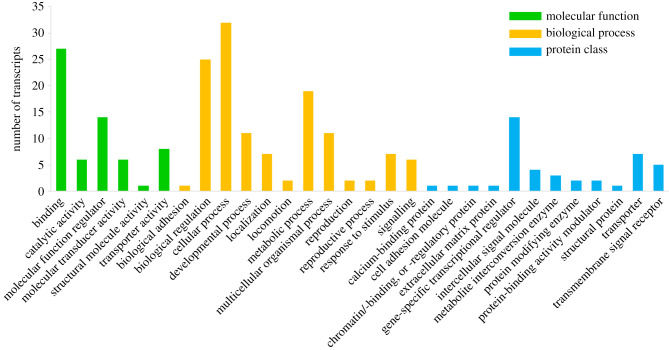
GO annotation in head-specific DEGs. PANTHER analysis was performed using 62 optimized DEGs annotated from the Uniprot database for functional annotation. The bar chart shows the distribution of genes within each representative group (molecular function, biological process, protein class).

Gene ontology (GO) terms associated with DEGs were used to identify differences within representative classification (molecular function, biological process, protein class) (figure 3). Genes related to binding (GO:0005488), cellular process (GO:0009987) and gene-specific transcription factor (PC00264) accounted for the highest at 43.5% (27/62), 51.6% (32/62) and 22.6% (14/62), respectively. Interestingly, among these genes, homeodomain transcription factors (OTX1, SIX3, SIX6, Nkx1–2, Hmx) were the most common, followed by basic helix–loop–helix transcription factors (SIM1, SIM2, PTF1A, FER3). These results indicate that the expression of common anterior developmental factors might be conserved in the anterior of leech embryos [3,38]. They also suggest that various transcription factors can activate crucial signalling pathways and give rise to organization of the nerve system in the anterior region [3,39–43].

For further investigation, we focused on genes that were both highly and specifically expressed in the head, assuming that highly expressed genes and those most differentially expressed would mainly contribute to head formation, including proboscis development. We selected carefully based on criteria (highly expressed genes annotated, head fragments per kilobase of transcript per million mapped reads (FPKM) >100; highly expressed genes uncharacterized, head FPKM >100; differentially expressed genes annotated, head FPKM >10 and body FPKM <1), and 11 transcripts including three uncharacterized genes were identified (table 1). Among these head-related DEGs, genes encoding secreted frizzled-related protein (sFRP) homologues were highly and specifically expressed based on both criteria. These findings suggest that a variety of genes are related to the development of the anterior region. Among them, the high and characteristic expression of sFRPs might be correlated with anterior formation [3,38,44].

**Table 1.** Genes of interest selected based on the three criteria. Asterisks indicate previously studied genes (for detailed phylogeny, see electronic supplementary material, figure S2).

gene	expression level (FPKM)		*q*-value	source
	body	head		
highly expressed genes annotated (head FPKM >100)				
SFRP2 (sFRP1/2/5a*)	6.36	124.08	6.79 × 10^−6^	Uniprot-Swissprot
CML23	5.26	101.45	6.83 × 10^−6^	
HS71L	29.7	401.82	7.88 × 10^−5^	
SCOT1	15.97	116.86	4.61 × 10^−3^	
highly expressed genes uncharacterized (head FPKM > 100)				
UniRef50_T1FEH8	1.05	119.36	8.26 × 10^−11^	UniRef50
UniRef50_A7TSG4	16.24	182.23	3.04 × 10^−5^	
UniRef50_T1F354	51.5	246.87	4.90 × 10^−2^	
differentially expressed genes annotated (head FPKM >10 and body < 1)				
ZCH24	0.03	28.61	8.44 × 10^−14^	Uniport-Swissprot
DFP3	0.21	19.42	3.92 × 10^−9^	
DMTA2 (DMRT93B*)	0.41	22.09	1.17 × 10^−8^	
SFRP1 (sFRP1/2/5b*)	0.68	21.69	5.22 × 10^−8^	

In addition to transcriptional expression analysis, we also investigated spatial expression patterns of these 11 transcripts to examine their embryological contribution during organogenesis.

### 2.4. Widespread expression of the *hs71l* and *scot1* homologue

**Figure 4.**
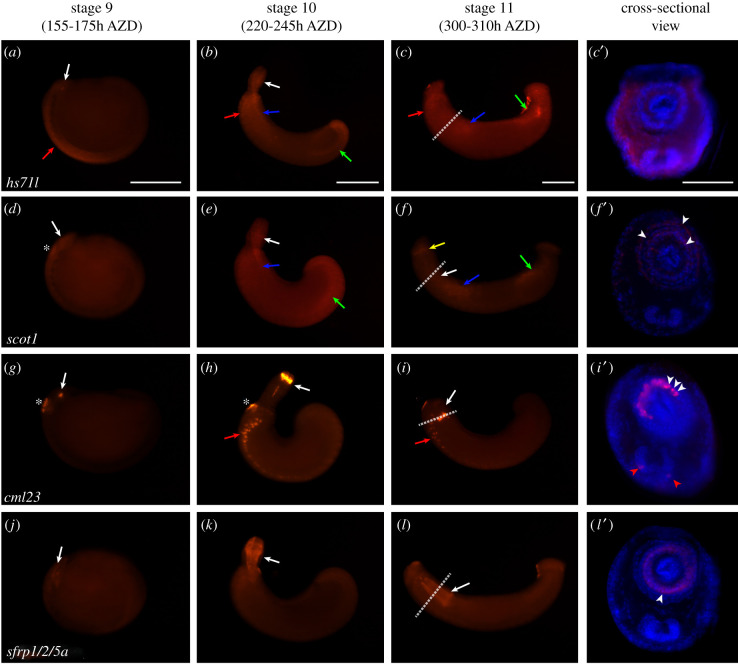
Spatial expression of the highly expressed gene (annotated) during organogenesis. Whole-mount *in situ* hybridization results of highly expressed genes (annotated). All embryos are oriented anterior to the left and dorsal to the top of the panel. The rightmost panel shows a cross-sectional view of a stage 11 embryo. White arrows indicate expression in the developing mouth part. Red arrows point to nervous expression of the candidate genes. Blue arrows indicate expression in the salivary gland. Green arrows point to the visceral muscle precursor. Yellow arrow indicates the expression in the intrinsic muscle. White asterisks indicate the expression in the adhesion site for attachment to the maternal venter. White dotted lines indicate the sectioned region seen in the rightmost panel. (*a*) *hs71l* transcripts are expressed in the stomodeum and the germinal plate. (*b*) *hs71l* expression is seen in the proboscis, rostral ganglia, salivary gland and developing posterior region involving the visceral muscle at stage 10. (*c,c′*) At stage 11, *hs71l* transcripts are expressed in the rostral ganglia, salivary gland and the developing hindgut, including whole mesodermal tissues in the body. (*d*) *scot1* is expressed in the adhesion site for attachment to the maternal venter and around the stomodeum. (*e*) At stage 10, *scot1* is expressed in the proboscis, salivary gland and posterior region involving the visceral muscle. (*f*,*f*′) At stage 11, *scot1* is expressed in the anterior intrinsic muscle, salivary gland and hindgut. In a sectioned view, its expression is found in the proboscis sheath, longitudinal muscle and radial muscle (white arrowheads). (*g*) At stage 9, *cml23* is expressed in the adhesion site for attachment to the maternal venter and in the stomodeum. (*h*) At stage 10, *cml23* is expressed in the apical proboscis, adhesion region and ventral ganglia. (*i*) *cml23* expression remains in the same region. (*i′*) In a sectioned view, *cml23* is expressed in cells of radial muscles (white arrowheads) and ventral ganglia (red arrowheads). (*j*) At stage 9, *sfrp1/2/5a* is expressed in the stomodeum. (*k*) At stage 10, *sfrp1/2/5a* is expressed in the radial muscle precursor in the everted proboscis. (*l*) *sfrp1/2/5a* expression is maintained at stage 11. (*l*′) *sfrp1/2/5a* is detected in the developing radial muscle (white arrowheads). Scale bar, 200 µm in panels *a*–*c*; 100 µm in panel *c*'. AZD, after zygotic development; *scot1*, succinyl-coenzymeA (CoA):3-ketoacid-CoA transferase 1; *cml23*, calmodulin-like 23; *sfrp1/2/5a*, secreted frizzled protein 1/2/5a.

In stage 9, a homologue of the heat shock protein (HSP) *hs71l* was expressed in the stomodeum and ventral germinal plate (figure 4*a*). Its expression in the mouthpart persisted. Its expression was also detected in the rostral ganglia, salivary gland and visceral muscle, including the precursor of the posterior sucker (figure 4*b*). After proboscis invagination, it was expressed ubiquitously in the whole body with discriminate expression in the rostral ganglia, salivary gland and hindgut (figure 4*c*-*c*′). HSPs are multifunctional proteins related to protein maturation, protein refolding, protein import and translocation. HSPs facilitate proteolytic degradation of unstable proteins by targeting proteins to lysosomes or proteasomes under normal and stress conditions [45–47]. Although some HSPs have been shown to have embryological functions such as neurodevelopment in previous studies [48,49], comparable expression patterns in lophotrochozoans have not to our knowledge been previously described. Our results for the first time suggest that *hs71l* plays multifunctional roles in tissues or organs undergoing dynamic structural changes during leech embryogenesis.

Succinyl-CoA-3-oxaloacid CoA transferase 1 (SCOT1) is related to ketone metabolism, which catalyses acetoacetate to acetoacyl–coenzyme A (acetoacyl-CoA) in the mitochondria [50,51]. At stage 9, the *scot1* homologue was expressed around the stomodeum and in the adhesive zone, by which the embryo attaches to the maternal venter during the period of development after hatching from the fertilization membrane and cocoon and before the rear sucker becomes functional (figure 4*d*). At stage 10, *scot1* was expressed in the proboscis, salivary gland and visceral muscle (figure 4*e*). Even after proboscis invagination, its overall expression pattern in the proboscis persisted along with the developing head, salivary glands and hindgut (figure 4*f*,*f*′ and electronic supplementary material, figure S3). We believe that, during embryogenesis, *scot1* is expressed in structures and developing regions in which ketone metabolism might be required [52–54]. These results suggest that the stage of organogenesis might be a period in which tissues and organs undergo dynamic changes with organ-specific metabolic process of ketone.

### 2.5. Genes expressed in specific tissue layers in the proboscis

Previous lineage analyses [23,24,36] of leech proboscis development and our morphostructural analysis revealed that the development of the proboscis is marked by orderly expansion of radial muscles and differentiation of longitudinal muscles. We hypothesized that characteristic core signalling genes might contribute to this process.

Calmodulin (CaM) and calmodulin-like (CML) proteins known as major regulators of Ca^2+^-dependent signalling are key regulators of various mechanisms such as cell proliferation, motility and cell cycle progression [55–57]. In addition, CaM is an upstream regulator of calmodulin kinase (CaMK), which activates CaMK signalling to induce the development of the central nervous system [58,59]. At stage 9, transcripts of *cml23* homologue were expressed in the furrow of the stomodeum and the adhesion site for attachment to the maternal venter (figure 4*g*). The adhesion site develops into the future epithelium of the anterior sucker. Transcripts of the *cm123* homologue were continuously expressed in the region, including nuclei of radial muscle and ventral ganglia (figure 4*h*–*i*′). Our results indicate that the *cml23* homologue is related to the cellular process of the developing proboscis and central nerve system during organogenesis.

**Figure 5.**
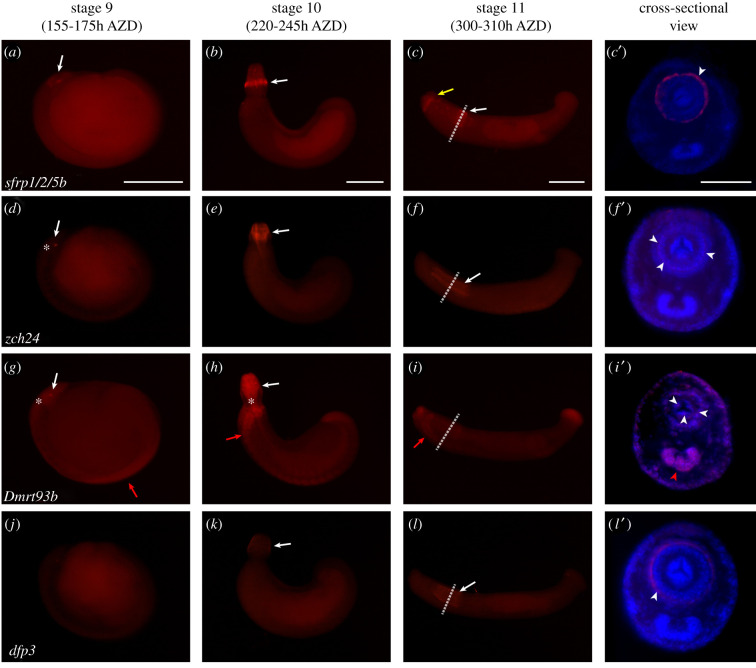
Spatial expression of DEGs (annotated) during organogenesis. Whole-mount *in situ* hybridization results of differentially expressed genes (annotated). All embryos are oriented anterior to the left and dorsal to the top of the panel. The rightmost column shows a cross-sectional view of a stage 11 embryo. White arrows indicate expression in the developing mouth part. Red arrows point to nervous expression of candidate genes. Yellow arrow indicates expression in the intrinsic muscle. White dotted lines indicate the sectioned region seen in the rightmost column. (*a*) By stage 10, *sfrp1/2/5b* is expressed in and around the stomodeum. (*b*) At stage 10, *sfrp1/2/5b* is expressed in the posterior epithelial tissues of the everted proboscis. (*c*,*c*′) After proboscis invagination, *sfrp1/2/5b* remains expressed in the proboscis sheath and the intrinsic muscle. (*d*) At stage 9, *zch24* is expressed inside the stomodeum. (*e*) After proboscis eversion, *zch24* is expressed in the radial muscle precursor. (*f*,*f*′) After proboscis invagination, *zch24* is expressed in the radial muscle layer (white arrowheads). (*g*) *Dmrt93B* is expressed in the ectodermal lineage in the germinal plate and the stomodeum. (*h*) *Dmrt93B* is expressed in the proboscis, ganglia and the visceral muscle region. (*i*) At stage 11, *Dmrt93B* expression remains in the rostral ganglia, proboscis and posterior sucker. (*i*′) In a cross-sectional view, *Dnrt93B* is detected in the epithelial tissue of the body, ventral ganglia (red arrowhead) and proboscis innervation (white arrowheads). (*j*) No *dfp3* expression is visible at stage 9. (*k*) At stage 10, *dfp3* transcripts start to be detected in the everted proboscis. (*l*,*l*′) By stage 11, *dfp3* is expressed in the longitudinal muscle layer (white arrowhead). Scale bar, 200 µm in panels *a*–*c*; 100 µm in panel *c*. AZD, after zygotic development; *sfrp1/2/5b*, secreted frizzled protein 1/2/5b; *zch24*, zinc finger CCHC domain-containing protein 24; *Dmrt93B*, doublesex- and male-abnormal-3-related transcription factor 93B; *dfp3*, defence protein 3.

sFRPs are key modulators of the Wnt signalling system, which determines the anteroposterior axis according to the distribution of Wnt ligands and their frizzled receptors; Wnt signalling is essential for bilaterian head formation [3,44]. In this study, *sfrp* orthologues showed an anterior-specific expression pattern in embryos during organogenesis (figures 4*j*–*l*′ and 5*a*–*c*′). At stage 9, these homologues were expressed around the stomodeum. At stages 10 and 11, these two paralogues showed different expression patterns. Transcripts of *sfrp1/2/5a* were expressed in the radial muscle precursor in the proboscis but weakly expressed in the posterior body (figure 4*k*–*l*′). However, *sfrp1/2/5b* was expressed in the posteriormost proboscis sheath and the anterior intrinsic muscle [60] (figure 5*b*-*c*′). Based on these spatial evidences, *sfrp* orthologues might play a role as conserved anterior morphogens with proboscis differentiation factors [3,44,61].

Zinc finger CCHC-type containing protein 24 (ZCH24) is one of the zinc finger domain proteins [62] known to be related to the development of somitogenic mesoderm in vertebrates [63], although it is largely unknown in invertebrates. In developing leech embryos, the *zch24* homologue was only expressed inside the stomodeum at stage 9 (figure 5*d*) and in radial muscle precursors from the anterior to the posterior of the proboscis by stage 11 (figure 5*e*–*f*′). These restricted expressions of *zch24* transcripts in proboscis indicate that *zch24* is a possible proboscis-specific gene in *H. austinensis* as well as related to the radial muscle differentiation of the developing proboscis.

### 2.6. Nervous system-specific expression of the *Dmrt93B* homologue

At stage 9, *Dmrt93B* was expressed around and inside the stomodeum and in the ectodermal region of the germinal plate (figure 5*g*). At stage 10, its transcripts were mainly expressed in the rostral segment of ganglia and the proboscis including ventral ganglia (figure 5*h*). After proboscis invagination, *Dmrt93B* transcripts were expressed in the rostral segments, epidermis, ventral ganglion, nerve canal in the proboscis cavity [16] and the posterior sucker (figure 5*i*,*i*′). *Dmrt* genes are well known to be mainly sex dimorphic factors [64,65]. They have been actively studied in various fields such as neuronal differentiation and brain development, in addition to differentiation of animal sex determinants and reproductive organs [66,67]. Furthermore, in Panarthropoda, *Dmrt93B* is expressed in the nervous system including mouth development [68]. Taken together, these results indicate that this highly conserved gene also plays a role as a transcription factor for the development of the mouth and nervous system during leech embryogenesis.

### 2.7. Developmental relevance of the defence-related gene

Defence protein is characterized by a reeler domain. It is an innate immune substance that protects against pathogens [69,70]. In this study, *dfp3* homologue was not expressed at stage 9 (figure 5*j*). However, it began to be expressed in the epithelium region of the proboscis at stage 10 (figure 5*k*). Its expression in the epithelium surrounding the proboscis was maintained even after proboscis invagination (figure 5*l*,*l*′). We suggest that this expression pattern is related to the development process of leech embryos. *Helobdella* embryos are surrounded by a vitelline membrane until stage 9. After hatching, they attach to the maternal venter and take off from it [25]. Based on the developmental evidence of spatial expression, the innate immune system in the leech might begin its protective function as the leech breaks through the vitelline membrane and becomes exposed to the environment.

### 2.8. Developmental contribution of uncharacterized genes during organogenesis

**Figure 6.**
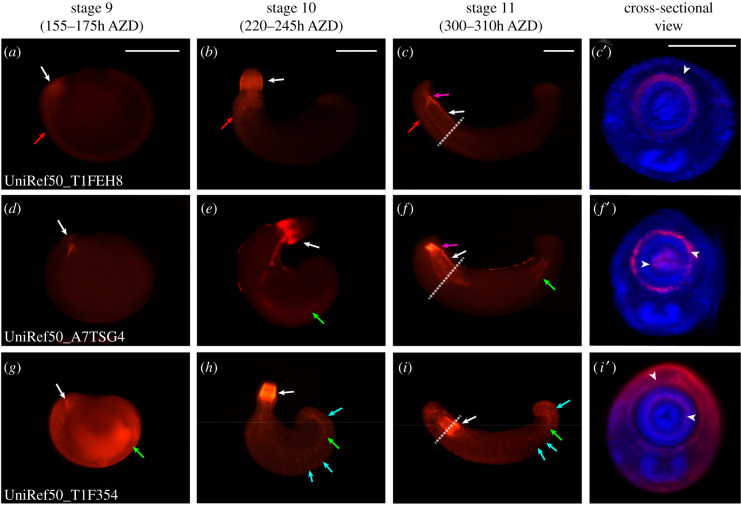
Spatial expression of the highly expressed gene (uncharacterized) during organogenesis. Whole-mount *in situ* hybridization results of highly expressed genes (uncharacterized). All embryos are oriented anterior to the left and dorsal to the top of the panel. The rightmost panel shows a cross-sectional view of a stage 11 embryo. Turquoise arrows indicate the somatic muscle fibres. Green arrows point to the visceral muscle precursor. Magenta arrows indicate the developing proboscis chamber. Red arrows point to nervous expression of candidate genes. White arrows indicate expression in the developing mouth part. White dotted lines indicate the sectioned region seen in the rightmost column. (*a*) At stage 9, transcripts of UniRef50_T1FEH8 are expressed broadly in the stomodeum and the anterior germinal plate. (*b*) At stage 10, UniRef50_T1FEH8 transcripts are expressed in the everted proboscis epithelium and rostral ganglia. (*c*,*c*′) At stage 11, the expression of UniRef50_T1FEH8 is detected in the longitudinal muscle and proboscis sheath region. (*d*) UniRef50_A7TSG4 is expressed in the stomodeum. (*e*) At stage 10, transcripts of UniRef50_A7TSG4 are expressed in the proboscis epithelium, oesophagus and gut boundary. (*f*,*f*′) At stage 11, transcripts of UniRef50_A7TSG4 are expressed in the developing hindgut, proboscis sheath and oesophageal funnel. (*g*) UniRef50_T1F354 is expressed in the whole body and strongly expressed in the coelomic cavity, visceral muscle and stomodeum. (*h*) At stage 10, UniRef50_T1F354 is expressed in the proboscis, muscle fibres of the body and visceral muscle. (*i*,*i*′) At stage 11, transcripts of UniRef50_T1F354 are expressed in the whole mesodermal tissue, including the proboscis sheath, radial muscle precursor and somatic muscle. Scale bar, 200 µm in panels *a*–*c*; 100 µm in panel *c*′. AZD, after zygotic development.

At stage 9, UniRef50_T1FEH8 transcripts were expressed in the periphery of the stomodeum along with overall expression in the germinal band Then, transcripts were expressed in the epithelium of the everted proboscis and rostral ganglia at stage 10. After proboscis invagination, UniRef50_T1FEH8 transcripts were expressed in the proboscis chamber, proboscis sheath and ventral ganglion (figure 6*a*–*c*′). From these results, we found that UniRef50_T1FEH8 is related to development of the epithelium of the proboscis and ganglia formation.

UniRef50_A7TSG4 transcripts were expressed in the foregut precursor region and around the stomodeum at stage 9 and in the proboscis epithelium, oesophageal funnel and overall gut boundaries at stage 10. At stage 11, the expression at stage 10 was maintained, in addition to the expression in the proboscis chamber, proboscis sheath, oesophagus and the developing hindgut (figure 6*d*–*f*′). Therefore, we found that UniRef50_A7TSG4 is related to the epithelium of the proboscis along with overall gut development.

UniRef50_T1F354 transcripts were expressed in the stomodeum, coelomic cavity and visceral muscle precursors at stage 9 (figure 6*g*). At stage 10, these transcripts were expressed in the radial muscle precursor in the anterior proboscis, fibres of the proboscis sheath, body and visceral muscles (figure 6*h*). The expression was maintained in the same region at stage 11 (figure 6*i*,*i*′). Therefore, UniRef50_T1F354 is related to the overall mesodermal tissue development during organogenesis. Their intense expression in the proboscis with major organs of these uncharacterized or maybe leech-specific genes indicates that these unique genes are not only important for embryogenesis but also are specifically related to the proboscis development.

## 3. Conclusion

The feeding organs of invertebrates have undergone extensive diversification and specialization over the course of evolution. We performed a comprehensive study to expand our knowledge about proboscis evolution and to discover genes related to the development of this specialized organ. Through transcriptome profiling, DEG analysis and spatial analysis, we identified specific genes related to proboscis development including highly conserved anterior formation factors. This not only provides novel information that has not been previously elucidated but also suggests that leeches also undergo anterior formation by conserved morphogens along with diverse transcription factors and that the developing proboscis is a vast hub of signal molecules. Furthermore, the presence of numerous uncharacterized genes in the anterior formation suggests a possible involvement of specific genes in proboscis development, including head formation in leeches, raising questions about the evolution of mouth formation in other proboscis-bearing animals. Taken together, our comprehensive survey will be used for comparative analysis of feeding organ diversity and proboscis formation in invertebrates.

## 4. Material and methods

### 4.1. Animals

Adult *H. austinensis* specimens were bred in the Laboratory of Cellular and Development Biology (Department of Biology, Chungbuk National University, Republic of Korea). They were bred in bowls containing artificial freshwater placed in a biological oxygen demand (BOD) incubator at 22°C. Embryos were placed in Petri dishes with lids containing *Helobdella triserialis* (HTR) saline medium (4.8 mM NaCl, 1.2 mM KCl, 2 mM MgCl_2_, 8 mM CaCl_2_ and 1 mM maleic acid) according to the protocol for handling leech embryos.

### 4.2. RNA isolation and RNA-seq library preparation

Embryos were cultured in clean HTR medium until stage 10 (220–245 h after zygotic development). To obtain a sufficient amount of RNA, about 400 embryos were used in the experiment. The head region was dissected using insect pins (Shiga, no. 2) under a Leica ZOOM 2000 stereomicroscope (Leica, Wetzlar, Germany). Next, the proboscis region was transferred to a 1.7 ml tube containing 200 µl of TRIzol reagent (Invitrogen, Carlsbad, CA, USA). RNA was isolated from each sample using TRIzol reagent according to the manufacturer's instructions. The purity and integrity of the total RNA isolated from dissected embryo samples were examined using a Nanodrop 2000C spectrophotometer (Thermo Scientific, Waltham, MA, USA) and a Bioanalyzer 2100 (Agilent Technologies, Palo Alto, CA, USA). Then, total RNA concentration was calculated using Quant-IT RiboGreen (Invitrogen, R11490). To assess the integrity of the total RNA, samples were run on a TapeStation RNA screentape (Agilent, 5067–5576). Only high-quality RNA (RNA integrity number greater than 7.0) was used to construct an RNA library. A library was independently prepared with 1 µg of total RNA for each sample using Illumina TruSeq Stranded Total RNA Sample Prep Kit v. 2 (Illumina, Inc., San Diego, CA, USA). Ribosomal RNA in total RNA was depleted using a Ribo-Zero kit (Epicentre Biotechnologies, Madison, WI, USA). After the rRNA depletion, the remaining RNA was purified, fragmented and primed for cDNA synthesis. Cleaved RNA fragments were copied into first-strand cDNAs using reverse transcriptase and random hexamers followed by second-strand cDNA synthesis using DNA polymerase I, RNase H and dUTP. Next, cDNA fragments underwent end repair, addition of a single A base and adapter ligation. Products were then purified and enriched with polymerase chain reaction (PCR) to create the final cDNA library. All libraries were quantified using quantitative PCR according to the *qPCR Quantification Protocol Guide* (KAPA Library Quantification kits for Illumina Sequencing platforms) and qualified using a TapeStation D1000 ScreenTape (Agilent Technologies, Waldbronn, Germany). Finally, library samples were sequenced using an Illumina HiSeq 2000 system (Illumina, Inc., San Diego, CA, USA) to generate paired-end reads.

### 4.3. *De novo* assembly, differential expression and gene ontology analyses

Transcriptome assembly was performed using Trinity v. 2.1.1 [71]. After assembly, protein-coding sequences (CDSs) within assembled transcripts were predicted using the Uniprot/Swiss-Prot database (http://www.uniprot.org), BLASTP v. 2.2.31+ and TransDecoder v. 3.0.0 (http://transdecoder.sourceforge.net) included in the Trinity software. Redundant CDSs with identity greater than 99% were clustered and the longest CDS was left using CD-HIT v. 4.6.5 [72]. Finally, a set of non-redundant coding sequences (NRCDS) was generated. To quantify each transcript's expression level, RNA-seq reads for each sample were aligned against the transcriptome sequences using Bowtie v. 2.2.6 [73] and abundances of reads according to transcripts were estimated with RSEM v. 1.2.26 [74]. For annotated NRCDS, homology search was performed against the Uniprot/SwissProt database using BLASTP with an *E*-value cut-off of 10^−10^ and the best blast hit. For one-to-one protein and gene mapping, the one with the highest expression level was selected if multiple NRCDS were annotated in a single gene. To minimize the loss of unannotated NRCDS, it was reannotated with the UniRef50 database, an automatically annotated database and clustered sets of sequences from UniProt Knowledgebase and selected UniParc records [37]. Differentially expressed genes between the head and body were identified using the edgeR program with an FDR cut-off of less than 0.05. GO terms of differentially expressed genes were identified using PANTHER (Protein Analysis Through Evolutionary Relationships, http://pantherdb.org/) [75].

### 4.4. Gene cloning and probe synthesis

Specific primers (electronic supplementary material, table S4) for genes of interest were designed based on transcriptome sequences. Genes were amplified from cDNA of *H. austinensis* at stage 10 using a TaKaRa E×Taq kit (Takara Bio Inc., Kusatsu, Japan) according to the manufacturer's instructions with the following cycling conditions: pre-denaturation at 94°C for 5 min, denaturation at 94°C for 30 s, variable annealing temperature for 30 s, variable extension time at 72°C and post-extension at 72°C for 5 min. These amplified fragments were cloned into pGEM T vector (Promega, Madison, WI, USA). RNAprobes labelled with digoxigenin were constructed using a MEGAscript kit (Ambion, Austin, TX, USA) and a DIG RNA Labeling Mix (Roche, Basel, Switzerland) according to each manufacturer's instruction.

### 4.5. Whole mount *in situ* hybridization

Fluorescence *in situ* hybridization (FISH) was performed as previously described [29,32] as follows: embryos were treated with protease from *Streptomyces griseus* (Sigma-Aldrich, St. Louis, MO, USA) in 1× phosphate-buffered saline (PBS), rinsed three times with glycine dissolved in PBS at room temperature (RT) for 5 min, fixed with 4% paraformaldehyde at RT for 40 min and rinsed with PBT (1× PBS+0.1% Tween-20) three times. Next, prehybridization was performed at 64.7°C for one day in hybridization buffer (50% formamide, 5× saline-sodium citrate (SSC), 1× Denhardt's solution, 0.1% 3-[(3-cholamidopropyl) dimethylammonio]-1-propanesulfonate (CHAPS), 100 mg ml^−1^ of heparin, 0.1% Tween 20 and 100 mg ml^−1^ of transfer RNA (tRNA)). After incubation, the prehybridized buffer was replaced with fresh hybridization buffer containing 2 ng ml^−1^ of corresponding probe. Embryos were then hybridized at 64.7°C for 2 days. After washing embryos with PBT, they were preincubated with maleic acid buffer (100 mM maleic acid, 150 mM NaCl, pH 7.5) at RT for 15 min, blocked with 1% blocking reagent for nucleic acid hybridization and detection (Roche, Basel, Switzerland) at RT for 2 h and incubated with anti-DIG/ POD (1/1000 dilution) in 1% blocking reagent at 4°C for 16 h. After incubation, embryos were rinsed twice with TNT buffer (0.1 M Tris–HCl (pH 7.5), 0.15 M NaCl, 0.1% Tween 20) at RT and washed with amplification solution provided by the NEN Tyramide Signal Amplification (TSA) Plus Kit (PerkinElmer, Wellesley, MA, USA). A colour reaction was initiated by adding a 1 : 50 dilution of reconstituted cyanine-3 tyramide in amplification solution. After checking the signal, the embryos were labelled with 4′,6-diamidino-2-phenylindole (DAPI) in PBT (1 : 100) at RT for 20 min in the dark. These embryos were imaged using a Leica DM6 B with a Leica DFC450 C camera (Leica, Wetzlar, Germany). Stained embryos were then dehydrated in ethanol, mounted in plastic embedding solution (PolyBed, Polysciences, Inc.) and used for sectioned images.

### 4.6. Immunostaining

Whole-mount immunostaining was performed according to previously published protocols [16]. Briefly, fixed embryos were washed with PBS three times. After washing with PBS, the embryos were rinsed with 1% Triton in 0.1 M Tris–HCl (pH 7.5) several times for 1 h, incubated with diluted block solution (1 : 9 = 10× Roche Western Blocking Reagent:PBT) for 2 h and then incubated with primary antibodies (anti-acetylated-α-tubulin produced in mouse; Sigma Aldrich; T-7451) in diluted blocking solution (1 : 500) at 4°C for 48 h. After five consecutive washes with PBT, embryos were incubated with a secondary antibody (goat anti-mouse IgG H&L Alexa Fluor 488, Abcam, ab150113) in diluted blocking solution (1 : 1000) at 4°C for 24 h. After checking the signal, embryos were washed five times with PBT and then stained with Texas Red-X Phalloidin (Thermo Scientific, Waltham, MA, USA) for 24 h to visualize F-actin. After checking the signal, embryos were washed with PBT five times and labelled with DAPI in PBT (1 : 100) at room temperature in the dark for 20 min. Fluorescence-stained embryos were imaged using a LSM 710 confocal microscope (Carl Zeiss, Oberkochen, BW, Germany). The obtained images were edited using ZEN software (Carl Zeiss). Fluorescence-labelled embryos were then dehydrated in ethanol series (70%, 90% and 100% diluted in 1 × PBS) and propylene oxide, followed by infiltration with plastic embedding solution (PolyBed). Embryos were cut with a microtome blade (Leica 818; Leica, Wetzlar, Germany) under an Olympus SZ-STS microscope (Olympus, Tokyo, Japan). These sections were then mounted with Fluoromount-G (SouthernBiotech, Birmingham, AL, USA). Stained embryos and slide samples were imaged using a LEICA DM6 B with a LEICA DFC450 C camera (Leica, Wetzlar, Germany). Obtained images were edited using a Las X software (Leica), and prepared as figure plates using Adobe Illustrator CS6 (Adobe, San Jose, CA, USA).

## Data Availability

Sequences generated in this study are deposited in GenBank (electronic supplementary material, table S4). Raw sequencing data are deposited in the NCBI Sequence Read Archive (SRA) database with accession numbers of SRR15013283 and SRR15013284 under BioProject PRJNA742851. The data are provided in electronic supplementary material [76].

## References

[1] Erwin DH. 2009 Early origin of the bilaterian developmental toolkit. Phil. Trans. R. Soc. B **364**, 2253-2261. (10.1098/rstb.2009.0038) 19571245 PMC2873006

[2] DuBuc TQ, Stephenson TB, Rock AQ, Martindale MQ. 2018 Hox and Wnt pattern the primary body axis of an anthozoan cnidarian before gastrulation. Nat. Commun. **9**, 1-12. (10.1038/s41467-018-04184-x) 29789526 PMC5964151

[3] Luo Y-J, Kanda M, Koyanagi R, Hisata K, Akiyama T, Sakamoto H, Sakamoto T, Satoh N. 2018 Nemertean and phoronid genomes reveal lophotrochozoan evolution and the origin of bilaterian heads. Nat. Ecol. Evol. **2**, 141-151. (10.1038/s41559-017-0389-y) 29203924

[4] Martinez Q, Lebrun R, Achmadi AS, Esselstyn JA, Evans AR, Heaney LR, Miguez RP, Rowe KC, Fabre P-H. 2018 Convergent evolution of an extreme dietary specialisation, the olfactory system of worm-eating rodents. Sci. Rep. **8**, 1-13. 30546026 10.1038/s41598-018-35827-0 PMC6293001

[5] DeMiguel D. 2016 Disentangling adaptive evolutionary radiations and the role of diet in promoting diversification on islands. Sci. Rep. **6**, 1-11. (10.1038/srep29803) 27405690 PMC4942836

[6] Lamichhaney S et al. 2015 Evolution of Darwin's finches and their beaks revealed by genome sequencing. Nature **518**, 371-375. (10.1038/nature14181) 25686609

[7] Tzetlin A, Purschke G. 2005 Pharynx and intestine. In Morphology, molecules, evolution and phylogeny in polychaeta and related taxa, pp. 199-225. Berlin, Germany: Springer.

[8] Resh VH, Cardé RT. 2009 Encyclopedia of insects. San Diego, CA: Academic Press.

[9] May RM. 1988 How many species are there on earth? Science **241**, 1441-1449. (10.1126/science.241.4872.1441) 17790039

[10] Barnes RD. 1987 Invertebrate zoology. Philadelphia, PA: WB Saunders Co.

[11] Sawyer RT. 1986 Leech biology and behaviour. Oxford, UK: Clarendon Press.

[12] Blanke A, Rühr PT, Mokso R, Villanueva P, Wilde F, Stampanoni M, Uesugi K, Machida R, Misof B. 2015 Structural mouthpart interaction evolved already in the earliest lineages of insects. Proc. R. Soc. B **282**, 20151033. (10.1098/rspb.2015.1033) PMC4528521 26203002

[13] Divers SJ, Mader DR. 2005 Reptile medicine and surgery (e-book). London, UK: Elsevier Health Sciences.

[14] Kapil S, Hendriksen S, Cooper JS. 2017 Cone snail toxicity. Treasure Island, FL: StatPearls Publishing. 29262115

[15] Göransson U, Jacobsson E, Strand M, Andersson HS. 2019 The toxins of nemertean worms. Toxins **11**, 120. (10.3390/toxins11020120) 30781381 PMC6410017

[16] Kwak H-J et al. 2021 Behavioral variation according to feeding organ diversification in glossiphoniid leeches (Phylum: Annelida). Sci. Rep. **11**, 1-13. (10.1038/s41598-020-79139-8) 34035418 PMC8149456

[17] Krenn HW. 2010 Feeding mechanisms of adult Lepidoptera: structure, function, and evolution of the mouthparts. Annu. Rev. Entomol. **55**, 307-327. (10.1146/annurev-ento-112408-085338) 19961330 PMC4040413

[18] Kingsolver J, Daniel T. 1995 Mechanics of food handling by fluid-feeding insects. In Regulatory mechanisms in insect feeding, pp. 32-73. Berlin, Germany: Springer.

[19] Kornev KG, Salamatin AA, Adler PH, Beard CE. 2017 Structural and physical determinants of the proboscis-sucking pump complex in the evolution of fluid-feeding insects. Sci. Rep. **7**, 1-18. (10.1038/s41598-017-06391-w) 28747640 PMC5529602

[20] Monaenkova D, Lehnert MS, Andrukh T, Beard CE, Rubin B, Tokarev A, Lee W-K, Adler PH, Kornev KG. 2012 Butterfly proboscis: combining a drinking straw with a nanosponge facilitated diversification of feeding habits. J. R. Soc. Interface **9**, 720-726. (10.1098/rsif.2011.0392) 21849382 PMC3284131

[21] Bauder JA-S, Handschuh S, Metscher BD, Krenn HW. 2013 Functional morphology of the feeding apparatus and evolution of proboscis length in metalmark butterflies (Lepidoptera: Riodinidae). Biol. J. Linn. Soc. **110**, 291-304. (10.1111/bij.12134) PMC4021108 24839308

[22] Gibson W, Peacock L, Hutchinson R. 2017 Microarchitecture of the tsetse fly proboscis. Parasites Vectors **10**, 1-9. (10.1186/s13071-017-2367-2) 28927459 PMC5606065

[23] Gline SE, Nakamoto A, Cho S-J, Chi C, Weisblat DA. 2011 Lineage analysis of micromere 4d, a super-phylotypic cell for Lophotrochozoa, in the leech *Helobdella* and the sludgeworm *Tubifex*. Dev. Biol. **353**, 120-133. (10.1016/j.ydbio.2011.01.031) 21295566 PMC3086575

[24] Huang FZ, Kang D, Ramirez-Weber F-A, Bissen ST, Weisblat DA. 2002 Micromere lineages in the glossiphoniid leech *Helobdella*. Development **129**, 719-732. (10.1242/dev.129.3.719) 11830572

[25] Weisblat DA, Kuo D-H. 2014 Developmental biology of the leech *Helobdella*. Int. J. Dev. Biol. **58**, 429. (10.1387/ijdb.140132dw) 25690960 PMC4416490

[26] Kim J-S, Kwak H-J, Jiménez BIM, Park SC, Xiao P, Weisblat DA, Cho S-J. 2017 Expression patterns of duplicated snail genes in the leech *Helobdella*. Dev. Genes Evol. **227**, 415-421. (10.1007/s00427-017-0598-z) 29188382

[27] Kim J-S, Jiménez BIM, Kwak H-J, Park SC, Xiao P, Weisblat DA, Cho S-J. 2017 Spatiotemporal expression of a twist homolog in the leech *Helobdella austinensis*. Dev. Genes Evol. **227**, 245-252. (10.1007/s00427-017-0585-4) 28699036 PMC5637724

[28] Kwak HJ, Ryu KB, Medina Jiménez BI, Park SC, Cho SJ. 2018 Temporal and spatial expression of the Fox gene family in the leech *Helobdella austinensis*. J. Exp. Zool. B: Mol. Dev. Evol. **330**, 341-350. (10.1002/jez.b.22828) 30280505

[29] Cho S-J, Vallès Y, Giani Jr VC, Seaver EC, Weisblat DA. 2010 Evolutionary dynamics of the wnt gene family: a lophotrochozoan perspective. Mol. Biol. Evol. **27**, 1645-1658. (10.1093/molbev/msq052) 20176615 PMC2912473

[30] Kang D, Huang F, Li D, Shankland M, Gaffield W, Weisblat DA. 2003 A hedgehog homolog regulates gut formation in leech (*Helobdella*). Development **130**, 1645-1657. (10.1242/dev.00395) 12620988

[31] Kuo D-H, Weisblat DA. 2011 A new molecular logic for BMP-mediated dorsoventral patterning in the leech *Helobdella*. Curr. Biol. **21**, 1282-1288. (10.1016/j.cub.2011.06.024) 21782437 PMC3152669

[32] Kwak H-J, Park J-S, Medina Jiménez BI, Park SC, Cho S-J. 2019 Spatiotemporal expression of anticoagulation factor Antistasin in freshwater leeches. Int. J. Mol. Sci. **20**, 3994. (10.3390/ijms20163994) 31426335 PMC6719055

[33] Graveley BR et al. 2011 The developmental transcriptome of *Drosophila melanogaster*. Nature **471**, 473-479. (10.1038/nature09715) 21179090 PMC3075879

[34] Olson PD et al. 2018 Genome-wide transcriptome profiling and spatial expression analyses identify signals and switches of development in tapeworms. EvoDevo. **9**, 1-29. (10.1186/s13227-018-0110-5) 30455861 PMC6225667

[35] Ruan J, Guo F, Wang Y, Li X, Wan S, Shan L, Peng Z. 2018 Transcriptome analysis of alternative splicing in peanut (Arachis hypogaea L). BMC Plant Biol. **18**, 1-11. (10.1186/s12870-017-1213-1) 29973157 PMC6032549

[36] Smith CM, Weisblat DA. 1994 Micromere fate maps in leech embryos: lineage-specific differences in rates of cell proliferation. Development **120**, 3427-3438. (10.1242/dev.120.12.3427) 21428108

[37] Suzek BE, Huang H, McGarvey P, Mazumder R, Wu CH. 2007 UniRef: comprehensive and non-redundant UniProt reference clusters. Bioinformatics **23**, 1282-1288. (10.1093/bioinformatics/btm098) 17379688

[38] Martín-Durán JM, Passamaneck YJ, Martindale MQ, Hejnol A. 2016 The developmental basis for the recurrent evolution of deuterostomy and protostomy. Nat. Ecol. Evol. **1**, 1-10. 10.1038/s41559-016-0005 28812551

[39] Kiecker C, Graham A, Logan M. 2016 Differential cellular responses to hedgehog signalling in vertebrates—what is the role of competence? J. Dev. Biol. **4**, 36. (10.3390/jdb4040036) 29615599 PMC5831800

[40] Wang W, Lo P, Frasch M, Lufkin T. 2000 Hmx: an evolutionary conserved homeobox gene family expressed in the developing nervous system in mice and *Drosophila*. Mech. Dev. **99**, 123-137. (10.1016/S0925-4773(00)00488-3) 11091080

[41] Segev E, Halachmi N, Salzberg A, Ben-Arie N. 2001 Nato3 is an evolutionarily conserved bHLH transcription factor expressed in the CNS of *Drosophila* and mouse. Mech. Dev. **106**, 197-202. (10.1016/S0925-4773(01)00437-3) 11472856

[42] Nambu JR, Franks RG, Hu S, Crews ST. 1990 The single-minded gene of *Drosophila* is required for the expression of genes important for the development of CNS midline cells. Cell **63**, 63-75. (10.1016/0092-8674(90)90288-P) 2242162

[43] Millen KJ, Steshina EY, Iskusnykh IY, Chizhikov VV. 2014 Transformation of the cerebellum into more ventral brainstem fates causes cerebellar agenesis in the absence of Ptf1a function. Proc. Natl Acad. Sci. USA **111**, E1777-E1786. (10.1073/pnas.1315024111) 24733890 PMC4035921

[44] Darras S et al. 2018 Anteroposterior axis patterning by early canonical Wnt signaling during hemichordate development. PLoS Biol. **16**, e2003698. (10.1371/journal.pbio.2003698) 29337984 PMC5786327

[45] Kim JY, Han Y, Lee JE, Yenari MA. 2018 The 70-kDa heat shock protein (Hsp70) as a therapeutic target for stroke. Expert Opin. Ther. Targets **22**, 191-199. (10.1080/14728222.2018.1439477) 29421932 PMC6059371

[46] Yoon YM, Kim HJ, Lee JH, Lee SH. 2019 Melatonin enhances mitophagy by upregulating expression of heat shock 70 kDa protein 1 L in human mesenchymal stem cells under oxidative stress. Int. J. Mol. Sci. **20**, 4545. (10.3390/ijms20184545) 31540288 PMC6769944

[47] Murthy VS, Ravishankar KV. 2016 Molecular mechanisms of heat shock proteins and thermotolerance in plants. In Abiotic stress physiology of horticultural crops, pp. 71-83. Berlin, Germany: Springer.

[48] Jagla T, Dubińska-Magiera M, Poovathumkadavil P, Daczewska M, Jagla K. 2018 Developmental expression and functions of the small heat shock proteins in *Drosophila*. Int. J. Mol. Sci. **19**, 3441. (10.3390/ijms19113441) 30400176 PMC6274884

[49] Miller DJ, Fort PE. 2018 Heat shock proteins regulatory role in neurodevelopment. Front. Neurosci. **12**, 821. (10.3389/fnins.2018.00821) 30483047 PMC6244093

[50] Fukao T et al. 2011 Clinical and molecular characterization of five patients with succinyl-CoA: 3-ketoacid CoA transferase (SCOT) deficiency. Biochim. Biophys. Acta (BBA)-Mol. Basis Dis. **1812**, 619-624. (10.1016/j.bbadis.2011.01.015) 21296660

[51] Newman JC, Verdin E. 2014 Ketone bodies as signaling metabolites. Trends Endocrinol. Metab. **25**, 42-52. (10.1016/j.tem.2013.09.002) 24140022 PMC4176946

[52] Cui W et al. 2019 Dysregulation of ketone body metabolism is associated with poor prognosis for clear cell renal cell carcinoma patients. Front. Oncol. **9**, 1422. (10.3389/fonc.2019.01422) 31921677 PMC6928137

[53] García-Caballero M et al. 2019 Role and therapeutic potential of dietary ketone bodies in lymph vessel growth. Nat. Metab. **1**, 666-675. (10.1038/s42255-019-0087-y) 32694649

[54] Linares A, Caamaño G, Diaz R, Gonzalez F, Garcia-Peregrin E. 1993 Utilization of ketone bodies by chick brain and spinal cord during embryonic and postnatal development. Neurochem. Res. **18**, 1107-1112. (10.1007/BF00966692) 8255360

[55] Chin D, Means AR. 2000 Calmodulin: a prototypical calcium sensor. Trends Cell Biol. **10**, 322-328. (10.1016/S0962-8924(00)01800-6) 10884684

[56] Toutenhoofd SL, Foletti D, Wicki R, Rhyner JA, Garcia F, Tolon R, Strehler EE. 1998 Characterization of the human CALM2 calmodulin gene and comparison of the transcriptional activity of CALM1, CALM2 and CALMS. Cell Calcium **23**, 323-338. (10.1016/S0143-4160(98)90028-8) 9681195

[57] Berchtold MW, Villalobo A. 2014 The many faces of calmodulin in cell proliferation, programmed cell death, autophagy, and cancer. Biochim. Biophys. Acta (BBA)-Mol. Basis Dis. **1843**, 398-435. (10.1016/j.bbamcr.2013.10.021) 24188867

[58] Wayman GA, Lee Y-S, Tokumitsu H, Silva A, Soderling TR. 2008 Calmodulin-kinases: modulators of neuronal development and plasticity. Neuron **59**, 914-931. (10.1016/j.neuron.2008.08.021) 18817731 PMC2664743

[59] Burkert P, Duch C. 2006 Developmental changes of CaMKII localization, activity and function during postembryonic CNS remodelling in *Manduca sexta*. Eur. J. Neurosci. **23**, 335-349. (10.1111/j.1460-9568.2005.04562.x) 16420442

[60] Brunet T, Fischer AH, Steinmetz PR, Lauri A, Bertucci P, Arendt D. 2016 The evolutionary origin of bilaterian smooth and striated myocytes. Elife **5**, e19607. (10.7554/eLife.19607) 27906129 PMC5167519

[61] Yamaguchi TP. 2001 Heads or tails: Wnts and anterior–posterior patterning. Curr. Biol. **11**, R713-R724. (10.1016/S0960-9822(01)00417-1) 11553348

[62] Aceituno-Valenzuela U, Micol-Ponce R, Ponce MR. 2020 Genome-wide analysis of CCHC-type zinc finger (ZCCHC) proteins in yeast, Arabidopsis, and humans. Cell. Mol. Life Sci. **77**, 3991-4014. (10.1007/s00018-020-03518-7) 32303790 PMC11105112

[63] Vitorino M, Correia E, Serralheiro A-R, De-Jesus A-C, Inácio JM, Belo JA. 2014 Expression pattern of zcchc24 during early *Xenopus* development. Int. J. Dev. Biol. **58**, 45-50. (10.1387/ijdb.130261jb) 24860994

[64] Kopp A. 2012 Dmrt genes in the development and evolution of sexual dimorphism. Trends Genet. **28**, 175-184. (10.1016/j.tig.2012.02.002) 22425532 PMC3350790

[65] Matson CK, Zarkower D. 2012 Sex and the singular DM domain: insights into sexual regulation, evolution and plasticity. Nat. Rev. Genet. **13**, 163-174. (10.1038/nrg3161) 22310892 PMC3595575

[66] Young FI, Keruzore M, Nan X, Gennet N, Bellefroid EJ, Li M. 2017 The doublesex-related Dmrta2 safeguards neural progenitor maintenance involving transcriptional regulation of Hes1. Proc. Natl Acad. Sci. USA **114**, E5599-E5607. (10.1073/pnas.1705186114) 28655839 PMC5514752

[67] Xu S, Xia W, Zohar Y, Gui J-F. 2013 Zebrafish dmrta2 regulates the expression of cdkn2c in spermatogenesis in the adult testis. Biol. Reprod. **88**, 11-12. 23175770 10.1095/biolreprod.112.105130

[68] Panara V, Budd GE, Janssen R. 2019 Phylogenetic analysis and embryonic expression of panarthropod Dmrt genes. Front. Zool. **16**, 1-18. (10.1186/s12983-019-0322-0) 31303887 PMC6604209

[69] Gandhe AS, Arunkumar K, John SH, Nagaraju J. 2006 Analysis of bacteria-challenged wild silkmoth, Antheraea mylitta (lepidoptera) transcriptome reveals potential immune genes. BMC Genom. **7**, 1-10. (10.1186/1471-2164-7-184) PMC1559613 16857061

[70] Qian C, Wang F, Zhu B-J, Wei G-Q, Sun Y, Li S, Wang L, Liu C-L. 2015 Identification and expression analysis of a novel defense gene from Actias selene. Orient. Insects **49**, 264-274. (10.1080/00305316.2015.1081648)

[71] Grabherr MG et al. 2011 Full-length transcriptome assembly from RNA-Seq data without a reference genome. Nat. Biotechnol. **29**, 644-652. (10.1038/nbt.1883) 21572440 PMC3571712

[72] Fu L, Niu B, Zhu Z, Wu S, Li W. 2012 CD-HIT: accelerated for clustering the next-generation sequencing data. Bioinformatics **28**, 3150-3152. (10.1093/bioinformatics/bts565) 23060610 PMC3516142

[73] Langmead B, Trapnell C, Pop M, Salzberg SL. 2009 Ultrafast and memory-efficient alignment of short DNA sequences to the human genome. Genome Biol. **10**, 1-10. (10.1186/gb-2009-10-1-r1) PMC2690996 19261174

[74] Li B, Dewey CN. 2011 RSEM: accurate transcript quantification from RNA-Seq data with or without a reference genome. BMC Bioinform. **12**, 1-16. (10.1186/1471-2105-12-1) PMC3163565 21816040

[75] Mi H, Thomas P. 2009 PANTHER pathway: an ontology-based pathway database coupled with data analysis tools. In Protein networks and pathway analysis, pp. 123-140. Berlin, Germany: Springer. 10.1007/978-1-60761-175-2_7 PMC6608593 19597783

[76] Kwak H-J, Lee S-G, Park S-C, Kim J-H, Weisblat DA, Park C, Cho S-J. 2022 Head transcriptome profiling of glossiphoniid leech (*Helobdella austinensis*) reveals clues of proboscis development. Figshare. 10.1098/rsob.210298 PMC8889196 35232253

